# Intelligent prediction of major adverse cardiovascular events (MACCE) following percutaneous coronary intervention using ANFIS-PSO model

**DOI:** 10.1186/s12872-022-02825-0

**Published:** 2022-08-30

**Authors:** Sahar Dehdar Karsidani, Maryam Farhadian, Hossein Mahjub, Azadeh Mozayanimonfared

**Affiliations:** 1 Department of Biostatistics, School of Public Health, Hamadan University of Medical Sciences, Hamadan, Iran; 2 Department of Biostatistics, Research Center for Health Sciences, School of Public Health, Hamadan University of Medical Sciences, P.O. Box 4171-65175, Hamadan, Iran; 3 Department of Cardiology, Medical School, Hamadan University of Medical Sciences, Hamadan, Iran

**Keywords:** CABG, Percutaneous coronary intervention, Major adverse cardiac events, Adaptive neuro fuzzy inference systems, Particle swarm optimization

## Abstract

**Background:**

This study aimed to use the hybrid method based on an adaptive neuro-fuzzy inference system (ANFIS) and particle swarm optimization (PSO) to predict the long term occurrence of major adverse cardiac and cerebrovascular events (MACCE) of patients underwent percutaneous coronary intervention (PCI) with stent implantation.

**Method:**

This retrospective cohort study included a total of 220 patients (69 women and 151 men) who underwent PCI in Ekbatan medical center in Hamadan city, Iran, from March 2009 to March 2012. The occurrence and non-occurrence of MACCE, (including death, CABG, stroke, repeat revascularization) were considered as a binary outcome. The predictive performance of ANFIS model for predicting MACCE was compared with ANFIS-PSO and logistic regression.

**Results:**

During ten years of follow-up, ninety-six patients (43.6%) experienced the MACCE event. By applying multivariate logistic regression, the traditional predictors such as age (OR = 1.05, 95%CI: 1.02–1.09), smoking (OR = 3.53, 95%CI: 1.61–7.75), diabetes (OR = 2.17, 95%CI: 2.05–16.20) and stent length (OR = 3.12, 95%CI: 1.48–6.57) was significantly predicable to MACCE. The ANFIS-PSO model had higher accuracy (89%) compared to the ANFIS (81%) and logistic regression (72%) in the prediction of MACCE.

**Conclusion:**

The predictive performance of ANFIS-PSO is more efficient than the other models in the prediction of MACCE. It is recommended to use this model for intelligent monitoring, classification of high-risk patients and allocation of necessary medical and health resources based on the needs of these patients. However, the clinical value of these findings should be tested in a larger dataset.

## Background

Coronary artery disease (CAD) as a common cause of death for both men and women worldwide has a significant global economic and health burden. It is estimated that more than 23.6 million deaths in various communities will be due to cardiovascular disease [1]. CAD is the first and most common cause of death in Iranians of all ages [2].

Percutaneous coronary artery bypass grafting (PCI) is one of the most common treatments for CAD, which has significantly reduced disability and mortality due to coronary heart disease by increasing survival rate [3, 4]. Despite the widespread use of PCI intervention as the most common form of myocardial revascularization, the possibility occurrence of major adverse cardiac events (MACE) has always been one of the challenges affects the prognosis of patients. Due to the increasing demand for this treatment, development of models to predict the occurrence of MACE as a clinical challenge, in line with the decision-making process for early intervention and treatment and rehabilitation programs for physicians and patients is essential [5–8].

Various studies have been conducted to develop models of predicting clinical outcome after PCI using classical statistical models such as logistic regression and machine learning methods. However, these PCI prediction models have focused mainly on in-hospital or short-term outcomes [9, 10]. Limited studies have been conducted on the development of predictive models based on machine learning algorithms for long-term PCI outcomes [11]. Also, the ambiguity and complexity of the relationships between variables and data imbalances is an important challenge to standard statistical approaches and reveals the need to use flexible usable prediction model in such situations.

The development of an expert system, especially an adaptive neuro-fuzzy inference system (ANFIS) method, over the past few decades has made crucial role in complex and uncertain medical tasks such as diagnosis and prediction of disease [12–14]. The most important advantages of these systems are: expression of human knowledge using special linguistic concepts and fuzzy rules, nonlinearity and compatibility, and better accuracy of these methods in terms of data constraints compared to other methods [15, 16]. Another new modeling method is artificial neural networks, the most important reason for the strength is their ability to be learned from input and output training patterns. The combination of fuzzy systems, which are based on logical rules and artificial neural networks with the ability to knowledge acquisition from numerical information, enables us to use human knowledge in the construction of the prediction model [17, 18]. In order to achieve more accurate results, one of the most important objectives when developing the ANFIS model is to update its parameters. Various methods such as genetic algorithm and particle swarm optimization method have been proposed to teach these parameters.

Therefore, this study aimed to predict the occurrence of long term major adverse cardiac (MACCE) events in patients undergoing stent angioplasty using the ANFIS model. Also, the predictive performance of ANFIS model was compared with ANFIS-PSO and logistic regression.

## Method

This retrospective cohort study comprised of 220 patients underwent PCI in Ekbatan Medical Center in Hamadan city, Iran from March 2009 to March 2012. From the beginning of September 2020 after at least 10 years of follow-up, the clinical condition of patients was followed up by telephone interview or in-person contact or by using the patients' medical records from the treating physician's office or hospital. This study has been approved by the Ethics Committee of Hamadan University of Medical Sciences with IR.UMSHA.REC.1398. 017.

Predictive models including ANFIS, PSO- ANFIS, and logistic regression were examined to predict the occurrence of MACCE. The odds ratio and 95% confidence interval were used to summarize and describe the data in the logistic regression model.

### Adaptive neuro-fuzzy inference system (ANFIS)

ANFIS as a fuzzy Takagi-Sugno system is based on if–then rules and benefits the learning skill of artificial neural networks together with decision-making capability of fuzzy-logic. The ANFIS structure is formed of five layers. The first layer performs the fuzzification process where the number and type of membership function are specified by a training system based on training data. Then, the rules are defined in the rule layer and the effect of each rule is calculated, which can also be called the inference layer. In the third layer, the effect of each rule is normalized according to the effect of other rules. For the fourth layer, the output of each rule is obtained, which calculates the weighted output. Finally, in the fifth layer, the outputs are added together to form the output of the fuzzy system. To create this network, several parameters such as the type of membership function, the number of functions, the learning method, and the number of repetitions (Epoch) must be optimized [19].

**Fig. 1:**
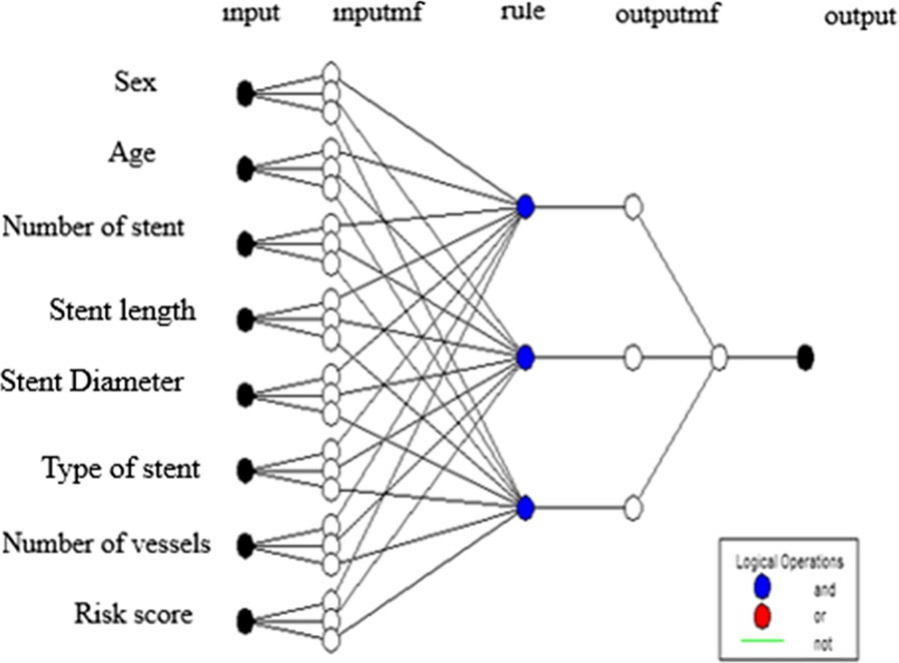
Proposed structure of the ANFIS model used in the present study

The Particle swarm optimization (PSO) is a stochastic optimization technique bio-inspired by the social behavior found in nature such as the motion of bird flocks and schooling fish. In this population-based algorithm, the members of the population interact straightly with each other and solve the problem by exchange of their best experience and recalling valuable experiences of the past. The beginning of the algorithm in optimizing the parameters of membership functions in the ANFIS model is that after designing the initial FIS, a group of particles or solutions are randomly generated and initialized by moving these particles during successive iterations and by updating the position of the particles, they try to find the optimal solution to the problem. For each answer, the fit value of each particle is evaluated, and if a better fit value is obtained, the position of the particle is updated. In the next step, the best new position of the whole group is found, and if the stop criterion is met, the algorithm also stops [15]. Proposed ANFIS architecture was shown in Fig. 1. ANFIS and ANFIS-PSO architecture and the training parameter presented in Table 1.

**Table 1:** ANFIS and ANFIS-PSO architecture and the training parameter

Fuzzy System	Sugeno
Membership function	Input: Gaussian Output: Linear
Training algorithm	back propagation
Max epoch	1000
Initial step size	0.01
Step size decrease rate	0.9
Step size increase rate	1.1
Error goal	0

Patients' baseline information including demographic characteristics, number of involved vessels, the number and type of stent (drug-eluting and bare-metal), stent size, height and length, history of smoking, history of diabetes, hypertension, and hyperlipidemia extracted from patient records. The risk score variable is defined as the sum of risk factors including diabetes, smoking, hypertension, and hyperlipidemia. If the person has the desired symptom, the value is 1, and if the symptom not exists, the value takes 0, so when the value of the risk score is 2 for an individual, it means that the person has 2 risk factors. The occurrence and non-occurrence of MACCE (including death, CABG, stroke, repeat revascularization) were considered as a binary outcome.

The input variables used in predictive models including sex, age, stent length, stent diameter, number of vessels, type of stent and risk score. For all models; the data were divided randomly into two groups of training (70%) and test (30%) sets. The same test, training sets and input variables were used to compare the performance of different models. Data analysis was performed using SPSS software version 21 and fuzzy logic toolbox of Matlab20 software. The stop criterion in the present study was a maximum of 1000 epochs or an error of less than 0.01. The best FIS was selected based on the lowest error rate.

## Results

At the end of the ten year follow-up time, out of 220 patients, 96 patients (43.6%) had experienced MACCE events. From those, 48 patients passed away (21.8%), 16 patients (7.3%) developed CABG, 5 patients had a non-fatal myocardial infarction (2.3%) and 27 patients (12.3%) required repeat revascularization. The results related to the distribution of different variables in terms of occurrence and non-occurrence of MACCE events are presented in Table 2.

**Table 2:** Frequency distribution of related variables in according to the occurrence of MACCE event

	Occurrence		Non-occurrence		Total	
Variable	N	%	N	%	N	%
All	96	43.6	122	56.4	220	100
Age (year)	Mean ± SD		Mean ± SD			*P*-value
	63.47 ± 1.05		57.31 ± 0.98			< 0.001
Sex	N	%	N	%	N	*P*-value*
Female	30	43.5	39	56.5	69	0.975
Male	66	43.7	85	56.3	151	
Smoking						0.001
Yes	39	61.9	24	38.1	63	
No	57	36.3	100	63.7	157	
Diabetes						< 0.001
Yes	31	77.5	9	22.5	40	
No	65	36.1	115	63.9	180	
Hypertension						0.008
Yes	47	54.7	39	45.3	86	
No	49	36.6	85	63.4	134	
Hyperlipidemia						0.041
Yes	31	55.4	25	44.6	56	
No	65	39.6	99	60.4	164	
Stent length						0.003
< 20 mm	51	36.2	90	63.8	141	
> 20 mm	45	57	34	43	79	
Stent diameter						0.784
3 mm	51	45.9	60	54.1	111	
3.5 mm	36	41.4	51	58.6	87	
4 mm	9	40.9	13	59.1	22	
Number of vessels						0.252
1	48	40	72	60	120	
2	31	44.9	38	55.1	69	
3	17	56.7	13	43.3	30	
Type of stent						0.577
BMS	60	42.3	82	57.7	142	
DES	36	46.2	42	53.8	78	
Number of stents						0.078
1	64	42.1	88	57.9	152	
2	23	41.1	33	58.9	56	
3	9	75	3	25	25	
Risk score						< 0.001
0	18	24	57	76	75	
1	29	40.8	42	59.2	71	
2	33	62.3	20	37.7	53	
3	16	76.2	5	23.8	21	

*Chi-square test risk score: SUM (Smoking, Diabetes, Hypertension, and Hyperlipidemia)

Based on the univariate analysis the mean age of patients with MACCE occurrence (63.47 ± 1.05) was significantly higher than those without the experience of MACCE (57.31 ± 0.98) (*p* < 0.001). No statistically significant difference was found in the distribution of MACCE events between the two sexes (*p* = 0.975). Approximately 43% of patients of both sexes had MACCE events. Smoking had a significant effect on the incidence of MACCE (*p* < 0.001), so that out of 63 smokers, 39 (63.9%) had MACCE, while out of 157 non-smokers, only 57 patients (36.3%) had experienced MACCE. Diabetes also had a significant effect on the MACCE (*p* = 0.001), from 40 diabetic patients, 31 (77.5%) had experienced MACCE, while out of 180 non-diabetic patients, 65 (36.1%) had experienced MACCE. Blood pressure and MACCE occurrence were also significantly associated (*p* = 0.008). Among 134 people who did not have high blood pressure, 49 (36.6%) had MACCE, while from 86 high blood pressure patients, 47 (54.7%) had MACE. The relationship between hyperlipidemia and the length of the embedded stent with MACCE occurrence was also statistically significant (*p* < 0.05). Variable such as stent number, stent type, stent diameter and the number of involved vessel had no significant effect on the MACE event.

In the multivariate analysis based on the logistic regression model presented in Table 3, the effect of age, smoking, diabetes, and stent length on the MACCE event was significant. In this regard, the results showed that a one-year increase at the age will increase the 5% chance of MACCE occurrence (95% CI: 1.02–1.09). Diabetes also increases the chance of developing MACCE by 5.77 times (95% CI: 2.05–16.202). The chance of developing MACCE in smokers is 3.5 times non-smokers (95% CI: 1.61–7.75). Also, the chance of MACCE occurrence in people who have a stent length greater than 20 mm is 3.12 times those in whom the stent length is less than 20 mm (95% CI: 1.48–6.57).

**Table 3:** Logistic regression analyses of factors associated with MACCE event

Variable	Adjusted odds ratio	95% CI	*P*-value
Age (year)	1.05	1.02–1.09	0.001
Sex			0.997
Female	Reference		
Male	0.99	0.44–2.22	
Smoking			0.002
No	Reference		
Yes	3.53	1.61–7.75	
Diabetes			0.001
No	Reference		
Yes	5.77	2.05–16.20	
Hypertension			0.25
No	Reference		
Yes	1.57	0.72–3.39	
Hyperlipidemia			0.238
No	Reference		
Yes	1.62	0.72–3.63	
Stent length			0.003
< 20 mm	Reference		
> 20 mm	3.12	1.48–6.57	
Stent diameter			0.316
3 mm	Reference		
3.5 mm	0.57	0.27–1.18	
4 mm	0.73	0.24–2.23	
Number of vessels			0.67
1	Reference		
2	1.02	0.37–2.74	
3	1.73	0.41–7.31	
Type of stent			0.99
BMS	Reference		
DES	1.002	0.47–2.13	
Number of stents			0.242
1	Reference		
2	0.84	0.38–1.85	
3	3.49	0.69–17.49	

**Fig. 2:**
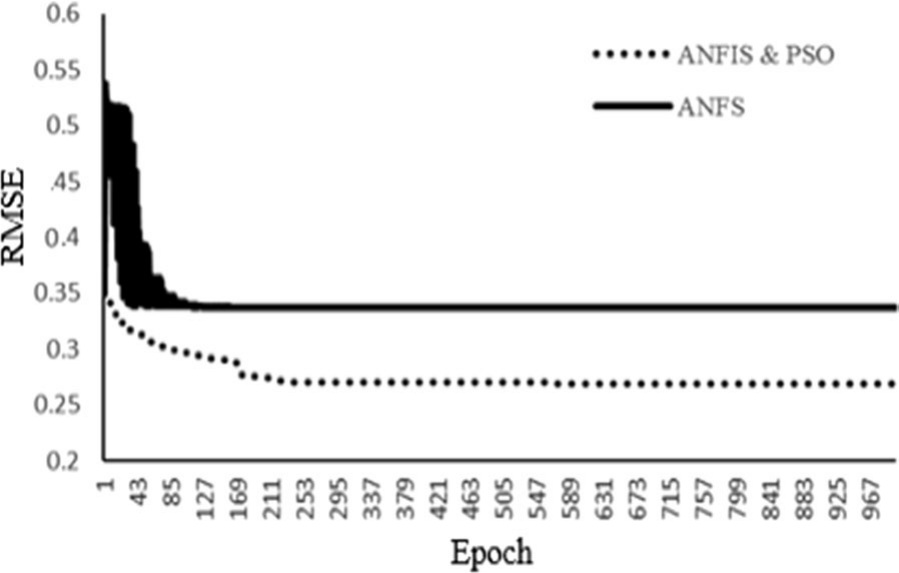
The rate of error change during the training process in the ANFIS and ANFIS-PSO model

**Fig. 3:**
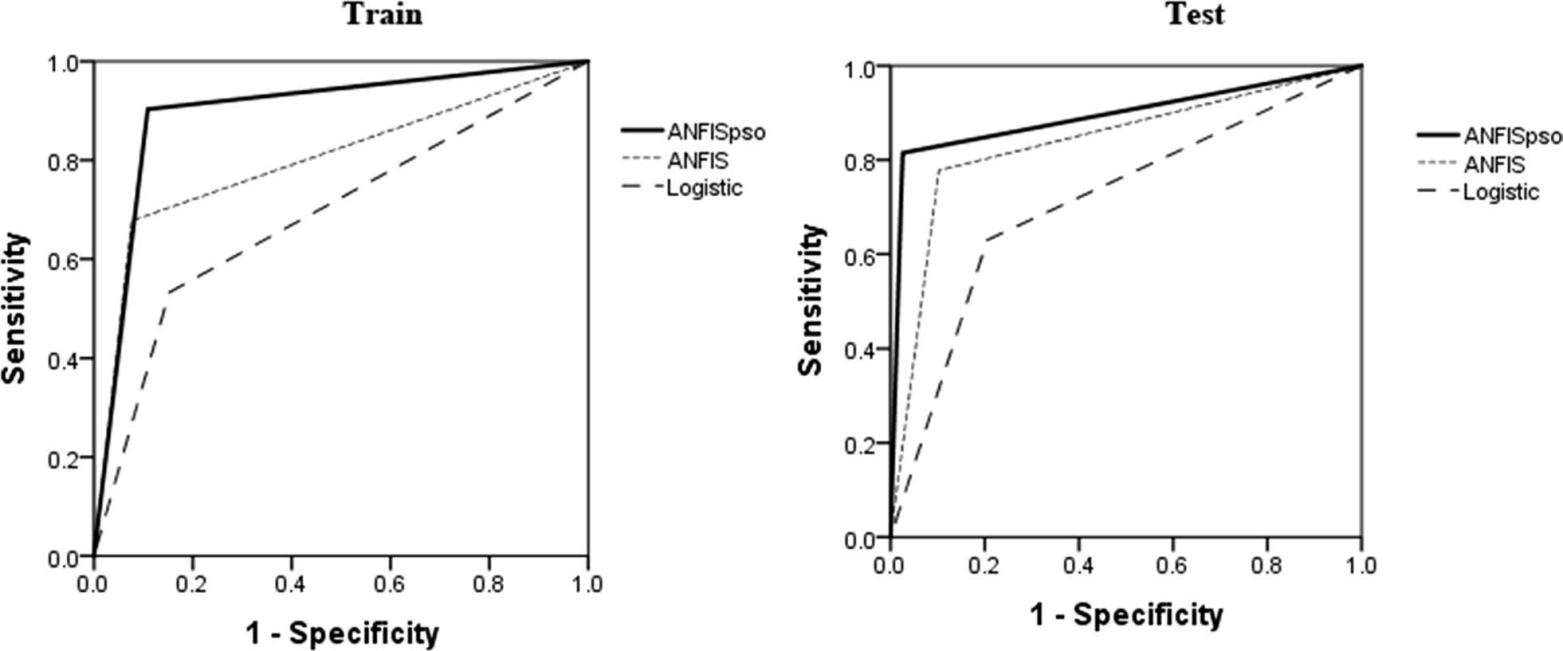
The area under the roc curve for the ANFIS, ANFIS-PSO and Logistic Regression on the train and test sets

The performance of the different predictive models in the training and test phase is presented in a Table 4. The process of error changes during the training process for the ANFIS and ANFIS-PSO models is presented in Fig. 2. Finally, a comparison of the prediction performance of three models in the form of sensitivity, specificity, accuracy and area under the roc curve (AUC) indices is presented in Table 4. Also, AUC plot for the models based on the train and test sets was presented in Fig. 3.

**Table 4:** Predictive performance of ANFIS, ANFIS-PSO and Logistic Regression on the test and train sets

Classification method	Train				Test			
	Accuracy	Specificity	Sensitivity	AUC	Accuracy	Specificity	Sensitivity	AUC
ANFIS	0.81	0.92	0.69	0.801	0.84	0.91	0.78	0.838
ANFIS-PSO	0.89	0.89	0.88	0.897	0.90	0.99	0.82	0.895
Logistic regression	0.72	0.85	0.53	0.690	0.71	0.79	0.62	0.712

## Discussion

In this study, the long-term (10-year) survival status of cardiovascular patients undergoing angioplasty was evaluated. Also, to predict the occurrence of MACCE, the performance of logistic regression model as the most common classical model, ANFIS and ANFIS-PSO as a machine learning model were compared.

One of the features of the present study is that it evaluates the long-term survival of patients, while other studies mainly consider short intervals (in hospital or 6 months follow up). Also, in these studies, the main focus has been on comparing the survival of patients with two types of drug-eluting and bare-metal stents. The present study was a retrospective cohort study in which no randomization was performed, so it was not possible to directly compare patient survival in two types of drug and metal stents. However, to modify its effect, this variable is added to the model.

The results show that 43.7% of patients experienced MACCE at the end of the follow-up period. While in the study conducted by Aghajani et al., the incidence of MACCE for 10-year follow-up was 14.4% [20]. In the study conducted by Spinola et al., based on the 313 patients over a ten-year period, 28% of these patients had experienced MACCE [21]. In the Meliga et al., study, which was conducted in seven American and European centers over 3-year follow-up period, MACCE events occurred in 26.5% of patients [22].

The multivariate results confirmed that the effect of variables such as age, smoking, diabetes and stent length was significant. The results indicate that the risk of MACCE in diabetic patients is significantly higher than non-diabetic patients (OR = 2.17). Similar results were observed by Aghajani et al. (OR = 1.33) [20], Meliga et al. (OR = 2.85) [21] and Cai et al. (OR 2.91) [23], Tsai et al. [24] and Farshidi et al. [25].

As expected, risk factors such as older age and smoking increase the risk of MACCE occurrence and are in accordance with the findings of Farshidi et al. and Tsai et al. [24, 25].

Also, patients with 3 stents implanted were 1.8 times more likely to have MACCE events than patients with 1 stent. This result was also confirmed in the Tsai et al. [25].

The ANFIS-PSO had a high accuracy in predicting the MACCE event compared to the ANFIS and the logistic regression models. Various studies have used classical and machine learning approaches to predict cardiovascular disease, for example: Taghizadeh et al., compared ANFIS and logistic regression in predicting death after coronary artery bypass graft surgery. Their study evaluated the data of 824 patients including age, sex, BMI, hypertension, diabetes (mellitus), blood cholesterol, peripheral vascular disease, addiction, smoking, history of chronic heart failure, etc. as input variables. The FCM method was used to create FIS model, Gaussian and linear membership functions were used for inputs and output. Sensitivity, specificity, accuracy were reported ANFIS 67%, 97% and 96% and in logistic regression 48%, 89% and 89%, respectively [26].

From the point of view of generalizability of the results of this study, considering that the information was collected retrospectively, it should be noted that some important basic characteristics have not been reported, such as early family history of CAD, BMI, race, culprit vessels, previous stroke, congestive heart failure, CKD and peripheral vascular disease. All of these variables can have a potential impact on the results of interest.

## Conclusion

In recent years, due to the emergence of hybrid prediction methods that help in screening and predicting the consequences of the disease, diagnosis and prediction in the field of medicine has made significant progress. The predictive performance of ANFIS-PSO is more efficient than traditional ANFIS and logistic regression in the prediction of MACCE. Application of this model is recommended for intelligent monitoring, the classification of high-risk patients and the allocation of necessary medical and health resources based on the needs of these patients.

## Data Availability

The database used and analyzed in the present study is not publicly available as its information may compromise the participants' privacy and consent involved in the research. However, the data are available from the corresponding author, upon request.

## Abbreviations

ANFIS: Adaptive neuro-fuzzy inference system
CABG: Coronary artery bypass graft surgery
CAD: Coronary artery disease
DES: Drug-eluting stent
BMS: Bare-metal stents
MACE: Major adverse cardiac events
MACCE: Major adverse cardiac and cerebrovascular events
MI: Myocardial infarction
PCI: Percutaneous coronary intervention
PSO: Particle swarm optimization
